# A Review of Sleep and Its Disorders in Patients with Parkinson's Disease in Relation to Various Brain Structures

**DOI:** 10.3389/fnagi.2016.00114

**Published:** 2016-05-23

**Authors:** Isobel T. French, Kalai A. Muthusamy

**Affiliations:** Department of Surgery, University MalayaKuala Lumpur, Malaysia

**Keywords:** sleep, Parkinson's disease (PD), suprachiasmatic nucleus (SCN), non-rapid eye movement sleep (NREMS), rapid eye movement sleep (REMS), dopamine, hypocretin, REMS behavior disorder (RBD)

## Abstract

Sleep is an indispensable normal physiology of the human body fundamental for healthy functioning. It has been observed that Parkinson's disease (PD) not only exhibits motor symptoms, but also non-motor symptoms such as metabolic irregularities, altered olfaction, cardiovascular dysfunction, gastrointestinal complications and especially sleep disorders which is the focus of this review. A good understanding and knowledge of the different brain structures involved and how they function in the development of sleep disorders should be well comprehended in order to treat and alleviate these symptoms and enhance quality of life for PD patients. Therefore it is vital that the normal functioning of the body in relation to sleep is well understood before proceeding on to the pathophysiology of PD correlating to its symptoms. Suitable treatment can then be administered toward enhancing the quality of life of these patients, perhaps even discovering the cause for this disease.

## Introduction

**Figure 1:**
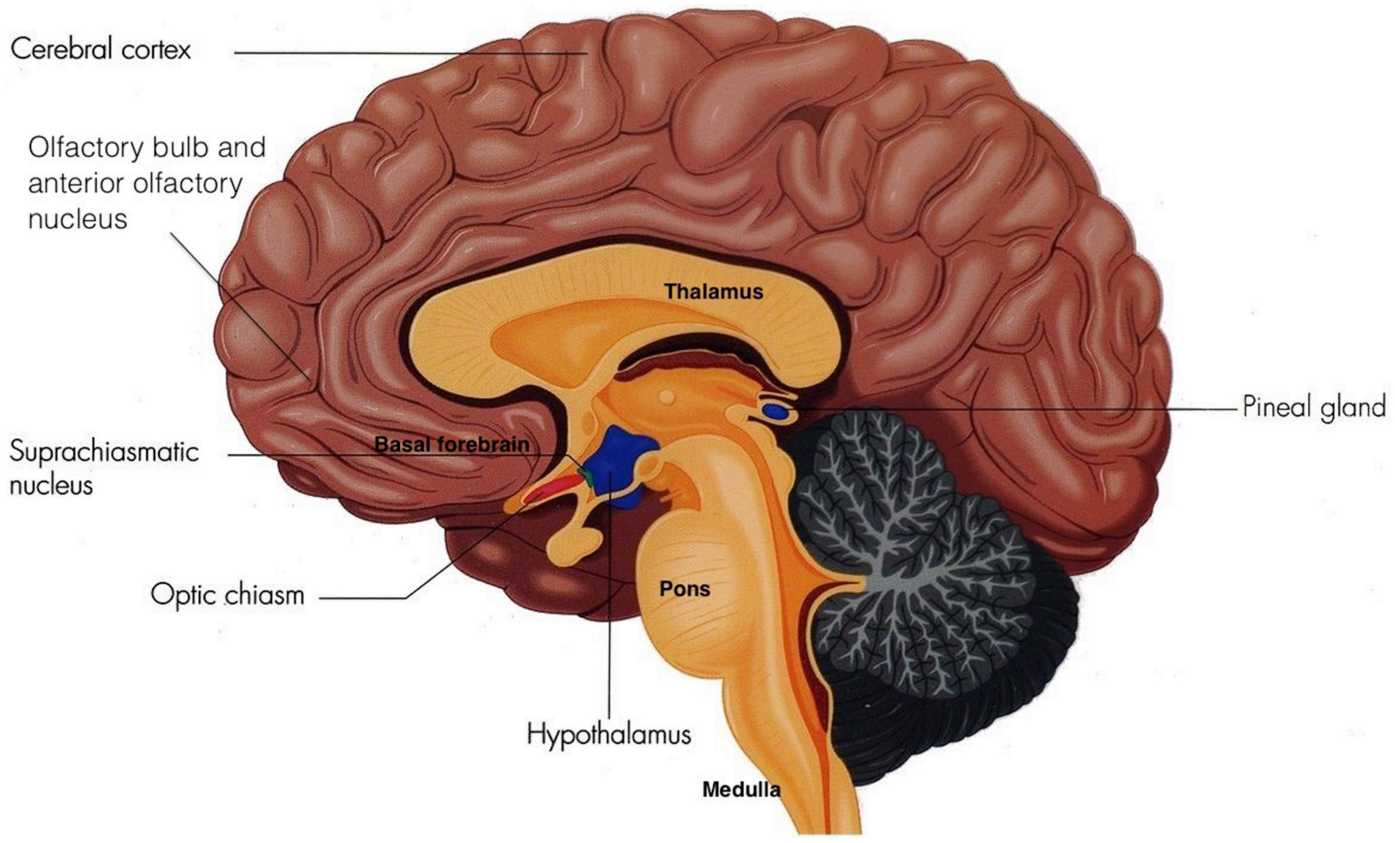
**The Suprachiasmatic Nucleus (Adapted and published under the Creative Commons Attribution-Share Alike 3.0 Unported license)**.

Sleep is vital to foster adequate health and bodily function (Altevogt and Colten, 2006) as it is an active physiological process needed to protect and normalize all major organs and regulatory systems (Altevogt and Colten, 2006; Barbara and Philips, 2006). Cellular function in the brain is altered radically during sleep (Maquet et al., 1997). Steadfast patterns of the aroused brain are substituted by low-frequency synchronization and fluctuating neuronal rhythms are displayed on the electroencephalogram (EEG; Niedermeyer and Lopes da Silva, 2005). The elementary mechanism of sleep is known as the sleep-wake cycle, which is controlled through homeostatic sleep drive (Process S) and circadian arousal drive (Process C; Altevogt and Colten, 2006; Barbara and Philips, 2006; NCBI Bookshelf, 2006; Moore, 2007). Process S induces sleep through its proposed regulator adenosine. Increasing wakefulness periods results in increased adenosine which increases the need for sleep (Barbara and Philips, 2006). Contrariwise, during sleep, levels of adenosine decrease, thus reducing the need for sleep. Circadian rhythms induce sleep through cyclical changes in the 24 h daily rhythms of physiology and behavior in an individual, which is regulated through the biological clock in humans (Barbara and Philips, 2006; NCBI Bookshelf, 2006). This biological clock consists of a pair of cluster neurons in the anterior ventral hypothalamus above the optic chiasm of the brain, known as the suprachiasmatic nucleus (SCN; see Figure 1).

Parkinson's disease (PD) is portrayed by a classical triad of movement disorders comprising bradykinesia, worsening resting tremors and rigidity with impaired postural reflexes (Williams-Gray et al., 2007). However, established non-motor symptoms also occur which lead to cognitive decline progressing onto dementia (Williams-Gray et al., 2007; Pontone et al., 2013). These include metabolic irregularities, altered olfaction, cardiovascular dysfunction, gastrointestinal complications, and, especially addressed in this review, sleep disorders and disturbances (Park and Stacy, 2009; Chaudhuri and Odin, 2010; Claassen et al., 2010; Menza et al., 2010; Ziemssen and Reichmann, 2010). An interesting aspect is that manifestation precedes decades before the onset of motor symptoms (Willison et al., 2013).

PD is thought to be caused by dopaminergic neuronal degeneration in the SN pars compacta (Garcia-Borreguero et al., 2003), which occurs due to Lewy body disease. This results in abnormal accumulations of α-synuclein in neurons. Obvious indicators are exhibited when sufficient nigral degeneration leads to the onset of motor symptoms (Boeve, 2013). However, it has become more illuminating as a multisystem disorder where numerous other brain structures are progressively affected as the disease advances (Jellinger, 2010; Jain, 2011; Willison et al., 2013). This is seen in the impaired functioning of the extrapyramidal system, which consequently damage arousal mechanisms (Arnulf, 2005) and thus ensues in sleep disorders.

## Sleep-wake regulation

Sleep-wake regulation is coordinated through the interchange of Process S and C (Moore, 2007), where process C functions to establish sleep and wake into discrete periods. Wakefulness is initiated and maintained through an activated cerebral cortex via two factors, where both arise from input from multiple activating systems. This is via mechanisms of the ascending reticular activating system (ARAS), and the resistance of Process S by Process C (Moore, 2007).

**Figure 2:**
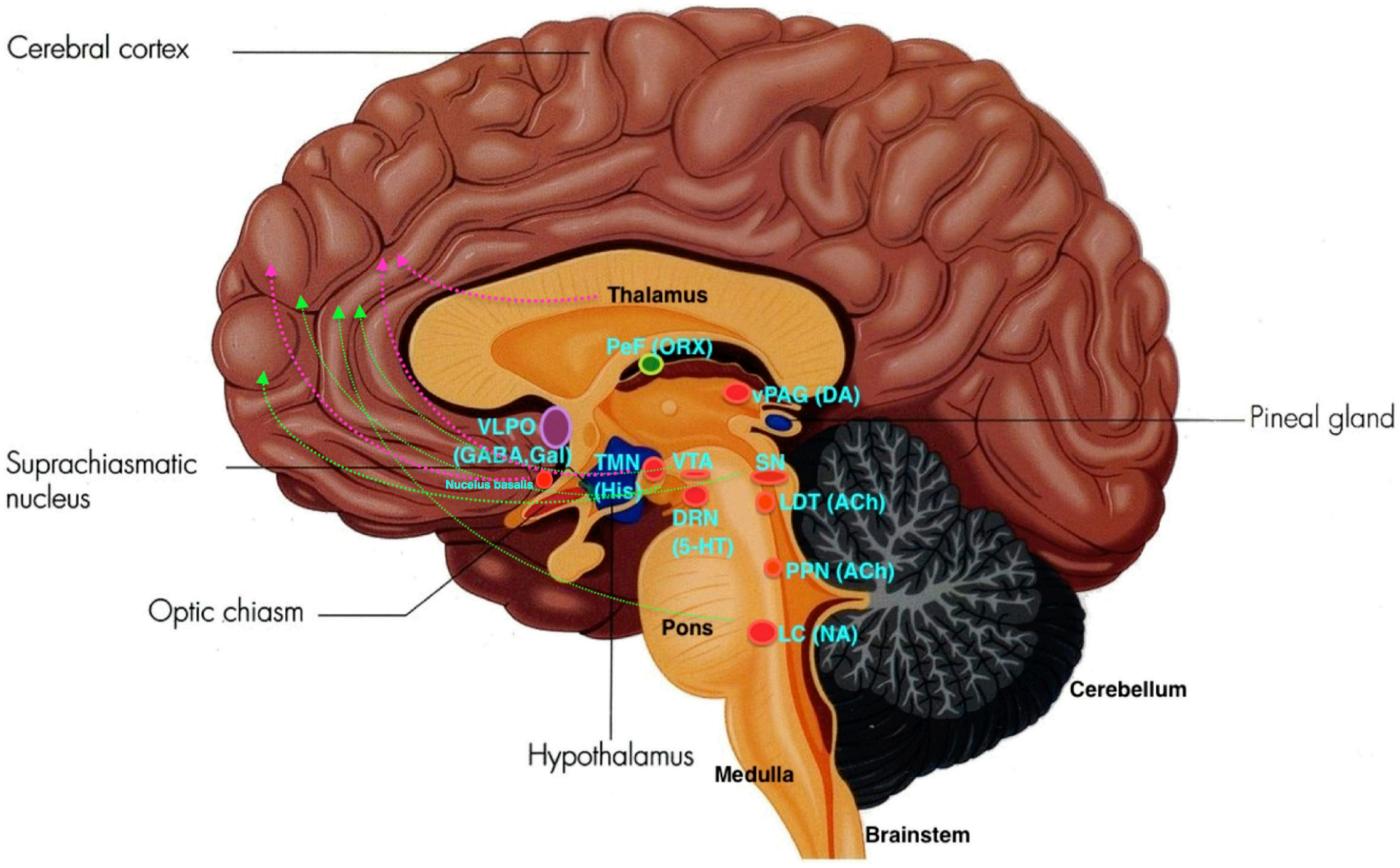
**The ARAS: VLPO, ventrolateral pre-optic nucleus; PeF, perifornical area; ORX, orexin; TMN, tuberomamillary nucleus; SN, substantia nigra; VTA, ventral tegmental area; DRN, dorsal raphe nuclei; SE, serotonin; LDT, laterodorsal tegmental nucleus; vlPAG, ventrolateral periaqueductal gray; PPN, pedunculopontine nucleus; LC, locus coeruleus; NE, norepinephrine**. Green lines depict the first pathway, and pink lines depict the second pathway. (Adapted and published under the Creative Commons Attribution-Share Alike 3.0 Unported license).

The ARAS from the brainstem activating the cerebral forebrain structures involves two major pathways. The first pathway includes projections from serotonin (SE) neurons of the dorsal raphe nuclei (DRN), noradrenaline or norepinephrine (NE) neurons of the locus coeruleus (LC), and dopamine (DA) neurons of the substantia nigra (SN) and ventral tegmental area (VTA). These neurons innervate the entire forebrain. The second pathway include projections from glutamate, hypocretin, and the histaminergic tuberomammillary nucleus (TMN) of the posterior hypothalamus, midline-intralaminar thalamus, and the cholinergic nucleus basalis neurons which innervate via the diencephalic and basal forebrain to the cortex (Moore, 2007; see Figure 2).

In PD, damage and cell loss occur in the dopaminergic VTA, serotonergic DRN, noradrenergic LC and vagus nerve dorsal nucleus cell, cholinergic Meynert basal nucleus, neocortex, hippocampus, Edinger Westpal and pedunculopontine nucleus (PPN), peptidergic neuropeptide Y neurons in the spinal cord, somatostatic hippocampus and cortex, substance P in the medulla, and the cholecystokinic and metenkephalic basal ganglia (Garcia-Borreguero et al., 2003; Arnulf, 2005). Significant reduction of hypocretin (Hcrt) is especially observed in the lateral cerebral ventricle of patients with severe PD (Arnulf, 2005).

## Sleep architecture in healthy adults

Sleep is divided into non-rapid eye movement sleep (NREMS), and rapid eye movement sleep (REMS; Walker, 2005) which alternate intermittently throughout the night (Altevogt and Colten, 2006; Barbara and Philips, 2006; Carskadon and Dement, 2011). NREMS is further divided into 4 stages (Walker, 2005), where stages 3 and 4 are collectively known as Slow Wave Sleep (SWS; Barbara and Philips, 2006).

A healthy adult falls asleep within 10 min and undergo a cyclic sequence of the five sleep stages, beginning with NREMS stage 1 advancing onto stage 2, followed by SWS and finally REMS. The individual then resumes back to NREMS (Carskadon and Dement, 2011), and the cycle recommences.

REMS and NREMS substitute throughout the night in an ultradian pattern every 85–100 min consisting of 3–5 cycles each night (Fry, 1985), where NREMS dictates the first half of the night mostly with SWS, whereas REMS and stage 2 NREMS abound in the latter half of the night (Walker, 2005).

### The sleep-wake cycle

Entry into NREMS stage 1 occurs when the circadian drive for arousal diminishes and Process S takes over Process C, where optimal adenosine levels are reached.

**Figure 3:**
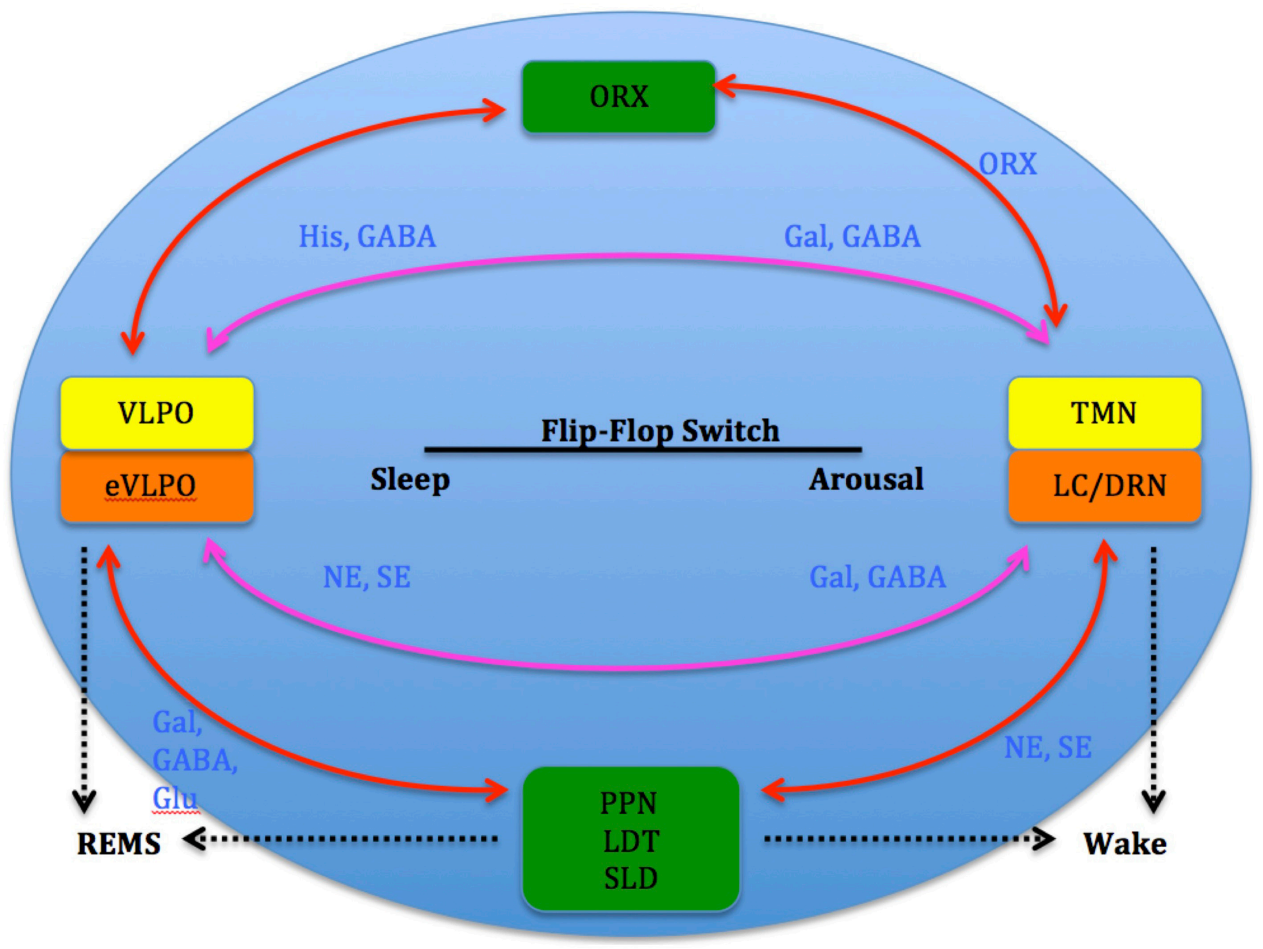
**Model depicting reciprocal interactions between sleep and wake promoting structures in the brain: VLPO, ventrolateral pre-optic nucleus; eVLPO, extended VLPO; ORX, orexin; TMN, tuberomamillay nucleus; LC, locus coeruleus; DRN, dorsal raphe nuclei, PPN, pedunculopontine nucleus; LDT, laterodorsal tegmental nucleus; SLD, sublaterodorsal nucleus; Gal, galanine; GABA, gamma-aminobutyric acid; NE, norepinephrine; SE, serotonin; His, histamine, Glu, glutamate**. (Saper et al., 2001).

Studies show that increases in adenosine or adenosine A1 receptor agonists in the rat basal forebrain and cat ventrolateral pre-optic nucleus (VLPO), promote sleep through inhibition of multiple wake-promoting brain areas and excitation of sleep-promoting cell groups (Scammell et al., 2000; Strecker et al., 2000). Adenosine also excites VLPO neurons by disinhibiting gamma-aminobutyric acid (GABA) inputs (Chamberlin et al., 2003). Thus, through inhibition of the basal forebrain arousal system and triggering of the VLPO nucleus, adenosine acts as a homeostatic regulator for sleep need, and induces sleep (Schwartz and Roth, 2008). Comprised of GABA and galanin (Gal), VLPO neurons inhibit arousal systems and set the thalamocortical network into a progressive state of synchronization, initiated by synchronous discharge of the thalamic reticular nucleus. This promotes sleep spindle generation and the initiation of stage 1 NREMS (Steriade, 2003; Llinás and Steriade, 2006; McCarley, 2007). The mechanism between activation of the VLPO and deactivation of the arousal systems is known as the flip-flop switch, which works to alter behavioral states between sleep and wake (see Figure 3).

Entry into stage 2 NREMS occurs when the thalamus begins generating and propagating sleep spindles (Schabus et al., 2007), which lead to the formation of k-complexes (Steriade, 2003). These measurements are generated from neuronal activity in the thalamic reticular nucleus, propagating through thalamic relay nuclei that traverse to the thalamocortical projecting nuclei in the cerebral cortex (de Andrés et al., 2011). Sleep spindles occur due to the frequency of these intermediate hyperpolarization through the interactions of GABA-activated thalamocortical and reticular thalamic nucleus neurons (McCarley and Chokroverty, 1994). K-complex waves (Izac and Eeg, 2006) occur when there is sudden increasing amplitude in these sleep spindles (Carskadon and Dement, 2011).

SWS initiates when brain activity decreases due to decline in cellular firing in most regions of the brain (J, 1995). This includes the forebrain which involves the association and motor cortex, the thalamic lateral geniculate nucleus and brainstem reticular formation, the DRN, and the LC. Decreases are also seen in the dorsal pons and basal ganglia (Maquet et al., 1997), reflecting significant deactivation of afferent thalamic nuclei and frontal cortical areas (Maquet et al., 1997). Exhibits of slow delta waves interspersed with smaller, faster waves are shown on the EEG (J, 1995). Activity decreases observed within the dorsal pons and mesencephalon reflects the persistent decrease in the firing rate of neurons of ascending brainstem systems. This leads to the hyperpolarization of the thalamic nuclei (Steriade and McCarley, 1990) which instantaneously generates SWS slow oscillations. However, increased firing is observed in portions of the amygdala and the hypothalamus. Since SWS displays decreased activity in the thalamocortical system and increased activity in portions of the limbic system, it is postulated to provide a rest period for skeletal muscles and optimize activity for the housekeeping systems of the internal body physiology (J, 1995).

Entry into REMS is shown on the EEG when intense brain activity shows either tonic activity, which is depicted by episodic bursts of rapid horizontal eye movements (Walker, 2005), or phasic activity, which is depicted by relative quiescence (Carskadon and Dement, 2011). REMS is generated by structures located in the lateral pontine region in the brainstem which performs as a command and control center (Siegel, 2000). It was originally proposed that REM-on and -off cellular activity governing REMS were cholinergic neurons. Activation of the reticular formation neurons would occur in a positive-feedback system and termination is induced through the inhibitory activity of REM-off aminergic neurons through REM-on activated neuronal modulation located within the laterodorsal tegmental nucleus (LDT) and PPN regions. Cholinergic activation of the cortex by the PPN then projects to the thalamocortical pathway to suppress slow delta waves and induce cortical arousal (Belaid et al., 2014). However, studies in rats show that brainstem glutamatergic and GABAergic neurons play a bigger role in producing and regulating REMS, particularly through REMS-on glutamatergic neurons located in the pontine sublaterodorsal tegmental nucleus (SLD). This is due to observations of a permanent glutamatergic input from the lateral and ventrolateral periaqueductal gray (vlPAG), and the removal of GABAergic inhibition at REMS onset which is present during wake and SWS. Nevertheless this does not preclude the role of cholinergic neurons in REMS regulation.

**Figure 4:**
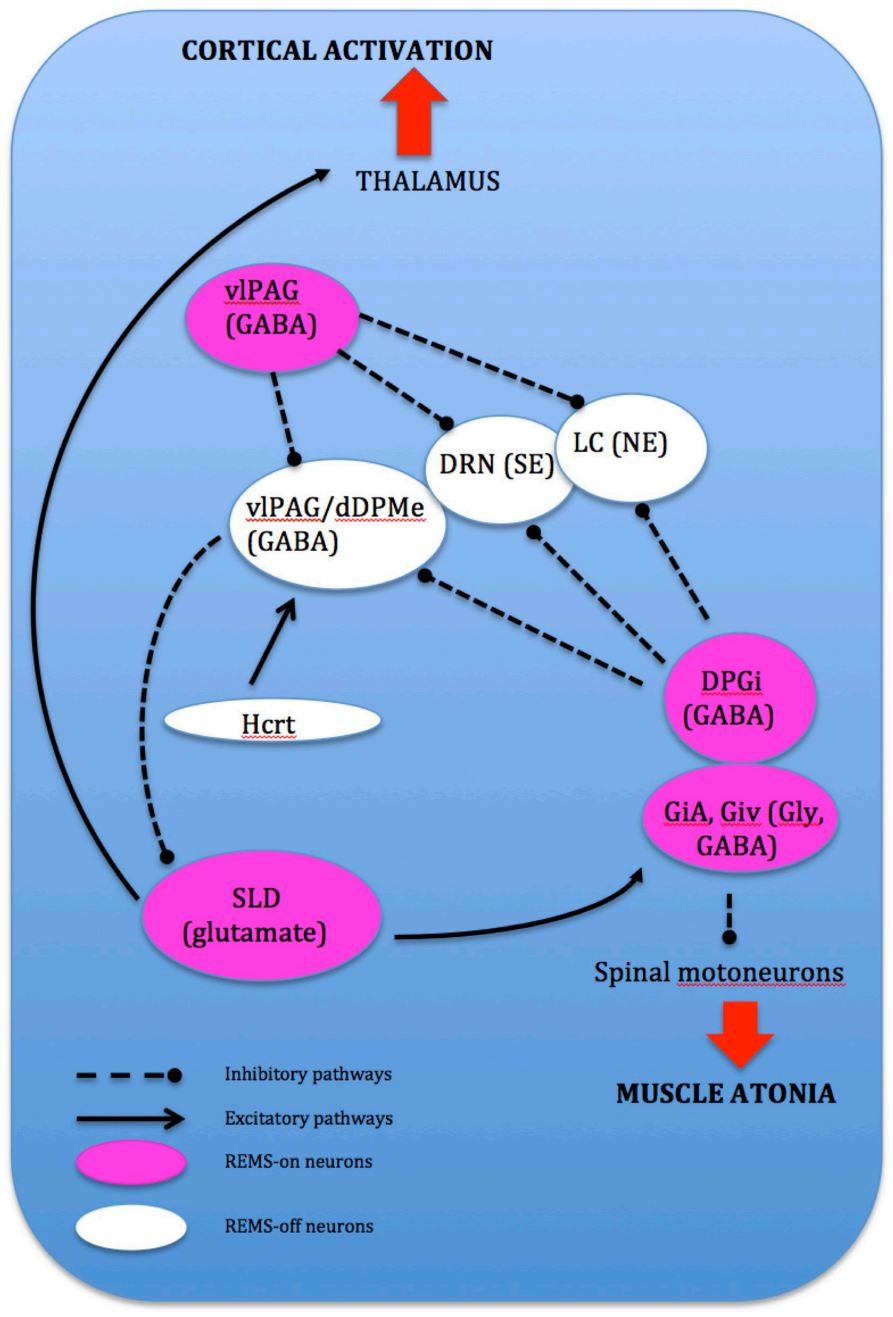
**Network model depicting REMS onset: DPGi, dorsal paragigantocellular reticular nucleus; dDpMe, dorsal deep mesenphalic reticular nucleus; DRN, dorsal raphe nucleus; GiV, ventral gigantocellular reticular nucleus; Hcrt, (orexin)-containing neurons; LC, locus coeruleus; vlPAG, ventrolateral periaqueductal gray; SLD, sublaterodorsal nucleus**.

REMS onset occurs when glutamatergic REMS-on neurons from the SLD are activated (Boissard et al., 2002). During wake and SWS, these REMS-on neurons are inhibited by a tonic inhibitory GABAergic tone derived from REMS-off neurons confined in the vlPAG and the dorsal deep mesenphalic reticular nucleus (Crochet et al., 2006). These neurons are activated during wake by hypocrtinergic and monoaminergic neurons (McGinty and Sterman, 1968). REMS onset is gated by activation of intrinsic mechanisms of REMS-on GABAergic neurons found in the dorsal paragigantocellular reticular nucleus and vlPAG (Gaus et al., 2002) as these neurons also inactivate the REMS-off monoaminergic neurons during REMS. The disinhibited ascending SLD PS-on neurons in turn induce cortical activation via projections to intralaminar thalamic relay neurons in collaboration with wake and REMS-on cholinergic and glutamatergic neurons from the LDT and PPN, the mesencephalic and pontine reticular nuclei, and the basal forebrain (Luppi et al., 2012). Descending REMS-on SLD neurons induce muscle atonia and sensory inhibition via excitatory projections to glycinergic pre-motoneurons localized in the alpha and ventral gigantocellular nuclei (McGinty and Sterman, 1968). Exit from REMS occurs when there is activation of the wake systems as REMS episodes are almost always terminated by an arousal. The wake systems then inhibit GABAergic REMS-on neurons localized in the DPGi and vlPAG (Figure 4).

Arousal occurs when the inhibitory interactions of sleep-promoting VLPO neurons and the NE, SE, and Ach neurons are down-regulated by the arousal systems which are blocked by the VLPO itself during sleep (Gallopin et al., 2000).

### The circadian system and the SCN

The circadian system consists of a network of central and peripheral oscillators throughout the brain and body, with its central clock located in the SCN (Willison et al., 2013). It is a component of the central autonomic network that controls autonomic functions in a state-dependent manner (Suzuki et al., 2011). The SCN modulates circadian oscillations in body systems such as body temperature, endocrine functions, blood pressure, sleep, and etc., through autonomous rhythms (Ropper, 2009).

**Figure 5:**
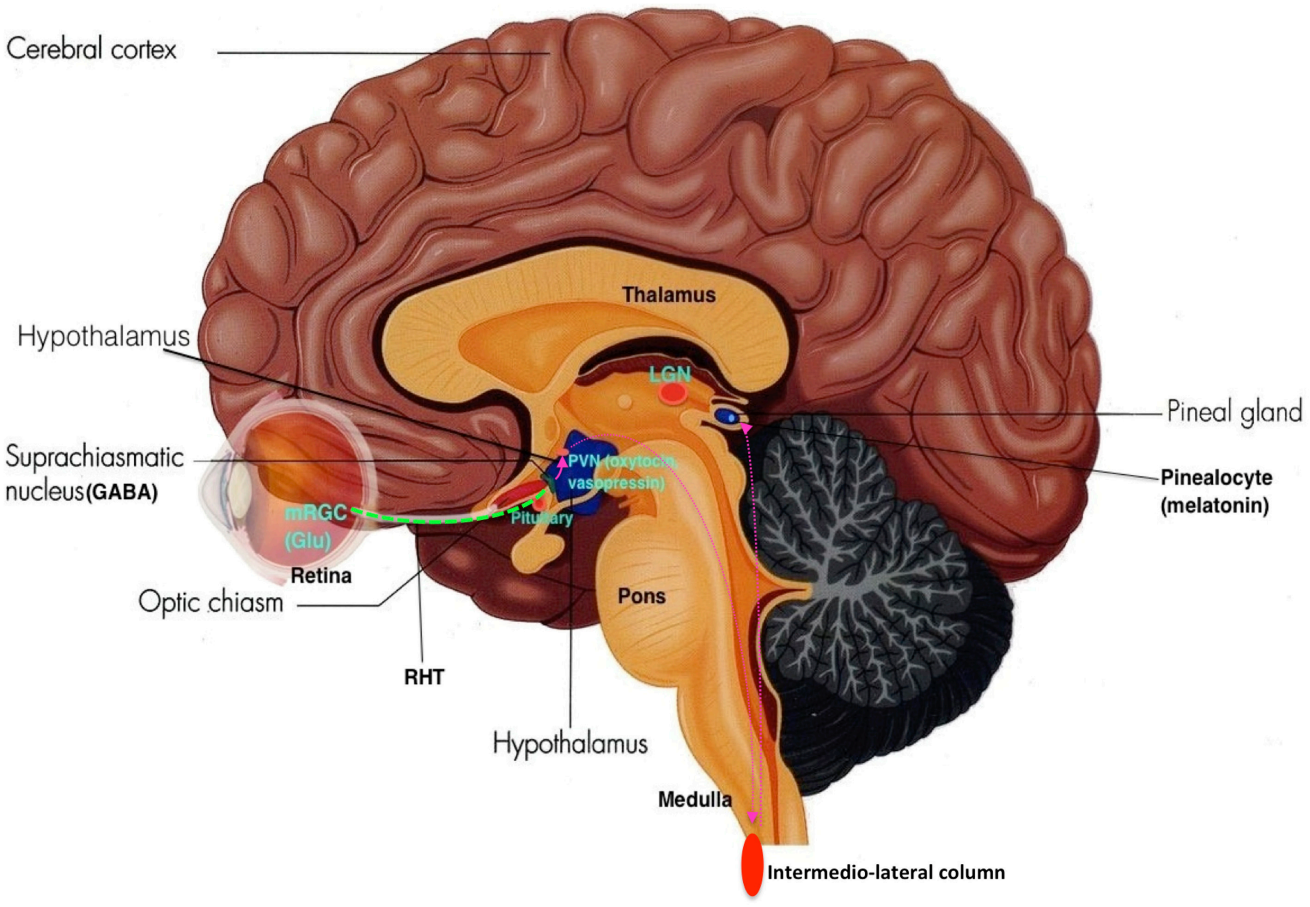
**The Retino-Hypothalamo-Pineal (RHP) Pathway (depicted by pink dotted lines): The retinohypothalamic tract (RHT) (green dotted line) is the first segment of the RHP pathway, which connects the eye to the hypothalamus**. Photons activate melanopsin-expressing retinal ganglion cells (mRGC) which project through the optic nerve to 2nd order neurons in the SCN. SCN neurons then project to 3rd order neurons in the paraventricular nucleus (PVN) of the hypothalamus. These neurons further project to 4th order preganglionic sympathetic neurons in intermediolateral zone of the thoracic lateral horns of the spinal cord. These neurons then project to 5th order postganglionic neurons in the superior cervical ganglia, which then project to 6th order cells (pinealocytes) in the pineal gland, thus making the neurohormone melatonin (Adapted and published under the Creative Commons Attribution-Share Alike 3.0 Unported license).

Neurons within the SCN receives light information from melanopsin-expressing retinal ganglion cells from the retina through the retinohypothalamic tract (RHT), where axons make direct synaptic connection with SCN cells. This photic information is assimilated with other timing cues to generate vigorous circadian oscillations synchronized to the environment, thus acting as a mechanism that entrains the circadian sleep-wake rhythm to the light-dark cycle of the natural environment (see Figure 5).

**Figure 6:**
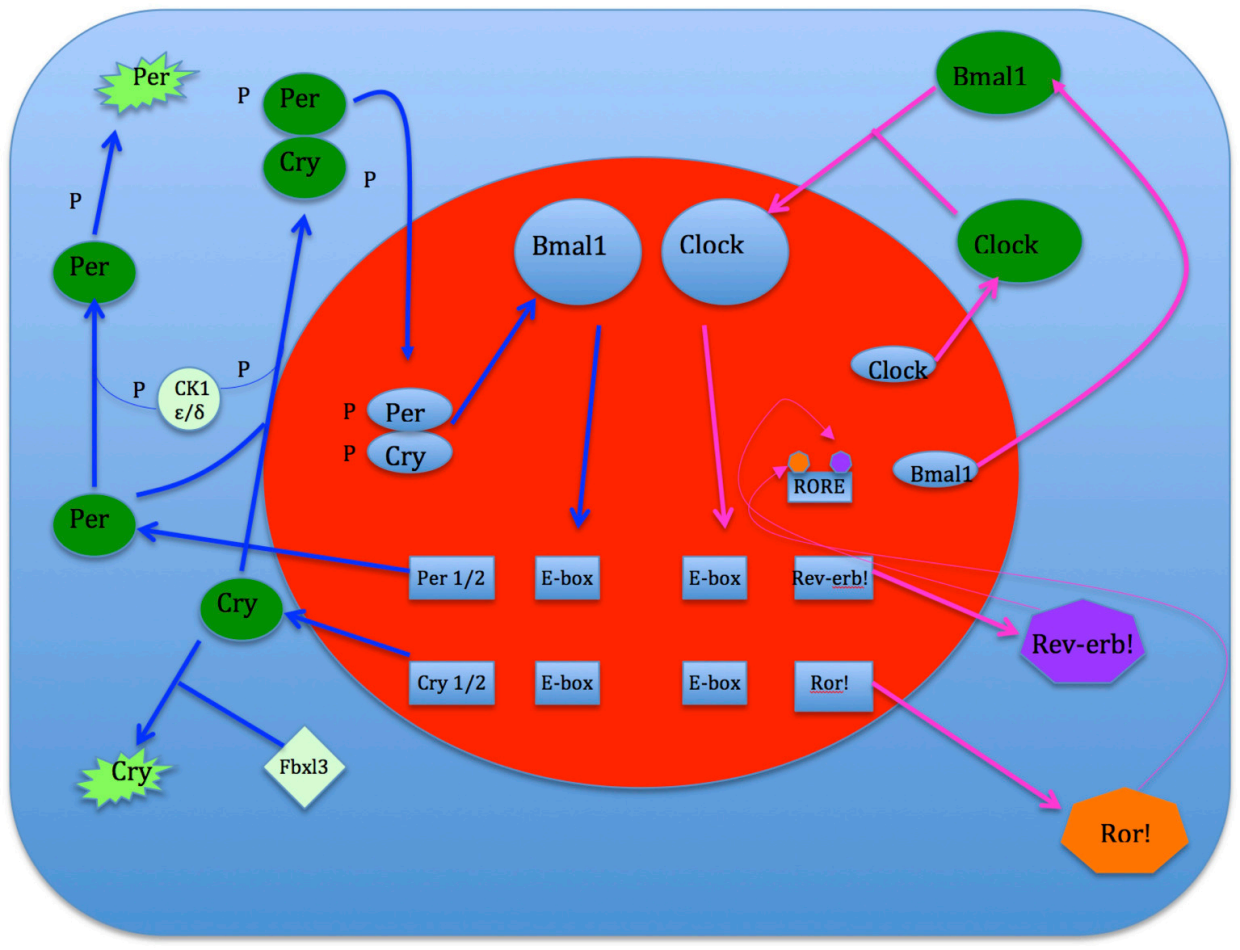
**Flip-flop switch model of sleep-wake regulation between VLPO sleep-promoting and arousal structures neurons (Saper et al., 2005)**.

Clock genes interact with each other to generate oscillations of gene expression, where successive gene activation is modulated in the form of a cycle. The initial activation of a gene is regulated by the last one in the sequence, making up an auto-regulatory feedback loop for which one cycle takes about 24 h. Positive components activate the expression of negative components, which reciprocally inhibit the activity of positive components. This system shifts the normal equilibrium and hence, continuous cycling results. The circadian clock mechanism consists of two interconnecting, regulatory feedback loops. The first loop regulates the transcription of Period (Per) and Cryptochrome (Cry 1,2). Two transcriptional activators, Bmal1 and Clock, form heterodimers in the cytoplasm and enter the nucleus where they bind to E-box sequences in the promoters of Per and Cry genes (Gekakis et al., 1998; Travnickova-Bendova et al., 2002; Etchegaray et al., 2003). This augments the expression activation of Per and Cry proteins which then interact with each other, enter the nucleus and inhibit the activity of Bmal1/Clock complexes (Kume et al., 1999; Sato et al., 2006). This sustains transcription activation levels of the Per and Cry genes. Thus Per and Cry genes shut off their own transcription when their activation levels decline (Ko and Takahashi, 2006). The second loop regulates the expression of the Bmal1 gene. In the nucleus Bmal1/Clock heterodimers bind to E-boxes in the promoters of genes that encode the retinoic acid-related orphan nuclear receptors, namely Rev-erb! And Ror!. These genes compete for the ROR element (RORE) in the Bmal1 promoter. Ror! activates Bmal1 expression, while Rev-erb! suppresses it. As a consequence oscillations of Bmal1 and Ror!/Rev-erb!are imbalanced. If activation overpowers expression, Bmal1 protein is produced and it forms heterodimers in the cytoplasm with Clock (Preitner et al., 2002). These heterodimers then enter the nucleus and initiate the next cycle of gene activation of both loops (see Figure 6).

Transport of clock proteins from the cytoplasm into the nucleus as well as posttranscriptional processes enhances regulation of the clock mechanism for generating oscillations of ~24 h (Harms et al., 2004). Interactions between Per and Cry proteins prevent rapid self-degradation and enables them to enter the nucleus. Interaction site mutation in either Per or Cry protein disturbs the nuclear and cytoplasmic localization with consequences on the clock oscillator (Ko and Takahashi, 2006). In the generation of mammalian circadian rhythms, phosphorylation and de-phosphorylation of Per proteins plays a critical role in determination of period length. The enzyme casein kinase 1ε or δ (CK1 ε/δ) phosphorylates Per2 protein at different sites. Thus mutations in the CK1ε as well as in sites of Per2 that are important for CK1ε binding and phosphorylation can cause alterations in period length (Lowrey et al., 2000).

DA is also an intermediary of light as it signals to the retinal circadian clock and provides direct input to the SCN (Witkovsky, 2004). Through dopaminergic amacrine cells in the retina, circadian rhythms are conveyed across core component clock genes of the molecular clock, including Per, Cry, Clock, and Bmal1. These genes help in tissue adaptation to environmental illumination to produce optimal photic response (Dorenbos et al., 2007). By utilizing dopaminergic D4 receptors, DA moderates rhythms in retinal second messengers like cAMP through direct interaction with retinal physiology and sensitivity (Jackson et al., 2011). DA also affects the phase and amplitude of specific clock genes through dopaminergic D1 receptors in the retina (Ruan et al., 2008).

Supplementary visual information is sent to the SCN by projections from the lateral geniculate nucleus, which is the thalamic relay for the primary visual system. The SCN then transmits signals to the hypothalamus, brain stem, pineal gland, and pituitary to facilitate the activity of sleep-wake systems of the brainstem as a primary circadian sleep-wake and bodily rhythm pacemaker (J, 1995).

SCN outputs massively innervate other hypothalamic regions, including the subparaventricular zone and other medial hypothalamic structures surrounding it. These hypothalamic relay nuclei innervate nervous and endocrine systems, where the SCN output transfers temporal information about the environment to the rest of the brain and body through multiple routes. Signals from the SCN also travel out via the hypothalamic-pituitary adrenal axis and the autonomic nervous system (ANS) to regulate independent circadian oscillators throughout the body (Dibner et al., 2010; Mohawk et al., 2012).

The SCN modulates major subcortical arousal centers including the LC and DRN which increase firing rate during the wake period (Aston-Jones et al., 2001; Deurveilher and Semba, 2005). Outputs from physiological systems that receive circadian regulation send feedback to modify and control SCN neural activity *in vivo* (Yamazaki et al., 1998; Schaap and Meijer, 2001; Deboer et al., 2003).

Another major feedback to the SCN is via the pineal gland, which regulates melatonin production and secretion through melatonin receptors located at a major site of the SCN. Melatonin is produced at high levels at night and low levels during the day, controlled by SCN projections to the autonomic subdivision of the paraventricular nucleus of the hypothalamus. The onset of sympathetic melatonin production and release inhibits firing of SCN neurons, which decreases SCN neuronal firing rate during late day and weakening the circadian drive (Process C) for arousal (Moore, 2007). This initiates Process S and the sleep-wake cycle.

### Circadian rhythm dysfunction in PD

The SCN is intact in PD patients however it functions through innervation with the hypothalamus and brainstem-associated central autonomic network.

The presence of defective pathways lead to malfunctioning effects (Willison et al., 2013), which lead to alterations in circadian rhythmicity. This reduces night-time sleep quality, daytime alertness and cognitive performance (Buysse et al., 2005).

In PD, a lack of time-dependent variation in Bmal1 expression in patients compared with controls were found (Breen et al., 2014). Patients were found to have abnormal sleep macro-architecture including increased sleep latency, reduced sleep efficiency, and reduced REMS. This is associated with certain circulating hormone profiles linked to peripheral clock gene expression differences. Such evidence suggests that sleep-wake disturbances in early PD reflects disorder in the neural circuitry controlling circadian rhythms. It is known that the hypothalamus is also affected in PD (Langston and Forno, 1978; Politis et al., 2008), which can lead to a decline in SCN activity responsible for reduced melatonin output and sleep-wake disruption (Wu and Swaab, 2005; Nakamura et al., 2011). Also, it was shown that mice overexpressing α-synuclein demonstrate reduced SCN firing rate, thus weakening the ability to communicate neural and hormonal signals from the central clock (Kudo et al., 2011). PD patients also showed a sustained elevation of serum cortisol levels and reduced circulating melatonin levels compared with elderly controls, similar to previous studies (Hartmann et al., 1997; Bordet et al., 2003).

A possible explanation for this is due to dysregulation in Bmal1 gene expression. Dopamine regulates Bmal1/ Clock heterodimer activity (Dibner et al., 2010), thus DA deficiency can directly affect this central component of the molecular clock. Striatal dopamine metabolism is apparently regulated by clock proteins such as Per2 (Hampp et al., 2008). Stimulation of dopamine receptors also affects the rhythm of clock gene expressions of Per1 and Per2 in the striatum (Imbesi et al., 2009; Hood et al., 2010). DA also regulates the rhythmic expression of melanopsin in retinal ganglion cells, thus influencing the entrainment of the circadian rhythm by light (Sakamoto et al., 2005). Considering that DA plays such a crucial role even in the circadian process of sleep, it should be highly plausible that the interconnecting relationships of different brain structures associated with the generation of sleep, including the SCN, can lead to different kinds of sleep disorders indefinitely. Alternatively, damage and degeneration to the SCN itself could be responsible for clock gene dysregulation, or even the hypothalamus. Whether circadian disruption in PD can be linked to the pathophysiology of other circadian sleep disorders such as advanced sleep phase syndrome (ASPS), delayed sleep phase syndrome (DSPS), non-24-h sleep wake syndrome, and seasonal affective disorder, poses a question as some of these disorders exhibit some of the symptoms seen in sleep disorders related to PD. Further research will be needed to know the exact mechanisms behind the occurrence of these disorders (Cermakian and Boivin, 2003).

The altered timing of physiological rhythms cause internal desynchronization, leading to loss of rhythm coordination which cause negative effects on rest-activity cycles and other physiological and behavioral functions (Reinberg and Ashkenazi, 2008). Circadian fluctuations also occur due to impaired retinal DA (Wirz-Justice et al., 1984).

Centrally, DA levels are modulated by monoamine oxidase A (MAO-A) and monoamine oxidase B (MAO-B). MAO-A is a clock-controlled gene (Hampp et al., 2008), thus disruption of the circadian clockwork due to loss of DA levels is expected to alter via MAO-A activity. Loss of DA neurons in PD therefore lead to circadian disruption as it alters sleep latency and desynchronizes diurnal rhythm changes (Willison et al., 2013).

Blood pressure, heart rate, cortisol and melatonin hormone levels are also altered due to autonomic dysfunction, and further lead to changes in sleep structure (Suzuki et al., 2011). The ANS is subject to circadian regulation, providing a balance between sympathetic and parasympathetic tone varying in synchrony with the daily circadian cycle (Jain and Goldstein, 2012). In healthy individuals, parasympathetic tone dominates during sleep to reduce heart rate and blood pressure through mechanics of the central circadian clock in the SCN via projections to the pre-autonomic neurons in the paraventricular nucleus (Buijs et al., 2003). The circadian regulation of the ANS is disrupted in PD patients, driving changes in blood pressure and heart rate (Kallio et al., 2000), thus changing sleep structure.

Anti-parkinsonian drugs also affect circadian rhythms and sleep-wake regulating systems either through direct action on sleep-wake regulating systems and circadian rhythm generators, or through indirect action meant to reduce PD symptoms during sleep (Garcia-Borreguero et al., 2003). It was found that the pineal gland undergoes compensatory up-regulation of monoaminergic transmitter systems outside of the basal ganglia, specifically in its uptake of L-DOPA, the pre-cursor of DA (Ghaemi et al., 2001). This was shown in a study conducted by Ghaemi et al. (2001) using the analog F-DOPA. The main factor contributing to this is long-term medication of L-DOPA, dopamine agonists or MAO-B inhibitors (Fowler et al., 1994). These drugs influence normal physiologic DOPA metabolism mechanisms of the pineal gland, which ultimately result in up-regulation of L-DOPA uptake. This is via up-regulation of the binding site of striatal DA uptake carriers found not only in the dopaminergic system, but adrenergic and serotonergic systems as well (Wiener et al., 1989). Thus dysregulation of pineal gland function not only arises from metabolism impairments in the dopaminergic systems, but from the adrenergic and serotonergic systems as well. This consequently leads to circadian rhythm dysfunction as the pineal gland is involved in melatonin synthesis which regulates the sleep-wake rhythm. Other evidence was found in a study which showed a nocturnal melatonin peak in PD patients undergoing treatment with L-DOPA which transpired earlier compared to de novo patients and healthy controls (Fertl et al., 1993).

### Sleep architecture in PD

PD patients experience reduced total sleep time and sleep efficiency, increased number of awakenings, and increased wakefulness after sleep onset (Wetter et al., 2000). Sleep fragmentation is the most consistent sleep disturbance, characterized by frequent awakenings and being awake 30–40% of sleep time. Some studies show a reduction in sleep spindles, SWS (Garcia-Borreguero et al., 2003), and REMS latency (Kendel et al., 1972; Poewe and Högl, 2000), whereas increased arousals lead to excessive daytime sleepiness (EDS). These symptoms reflect changes in the temporal pattern of sleep due to circadian dysfunction (Abbott et al., 2005; Dhawan et al., 2006; Reid and Zee, 2009; Mayer et al., 2011; Schulte and Winkelmann, 2011). Circadian disruption is thus a critical factor contributing to insomnia and hypersomnia in PD patients (Mohawk et al., 2012; Willison et al., 2013). PD patients also experience REMS behavior disorder (RBD) as structural impairments occur in the brainstem due to dopaminergic loss and pathway defects, which is the main region of the brain modulating REMS. Table 1 summarizes the different sleep disorders experienced in patients with PD. It shows the causes of each disorder and the structures affected, as well as its effect and symptoms.

**Table 1:** **Summary table of the different sleep disorders in PD, the cause and brain structures affected, and its effect and symptoms**.

**Sleep disorder type**	**Cause**	**Structures affected**	**Effect**	**Symptoms**
EDS	Arousal system damage	Orexin neuronal loss in posterior lateral hypothalamus VTA	More REM-on firing Narcolepsy	Sleep onset
		Impaired serotonergic (DRN), noradrenergic (LC) and cholinergic (PPN, LDT) neurons	Impaired wakefulness More REM-on firing	Sleep onset
	Dopaminergic drugs	D1/ D2 receptors	Sedation	Sleep onset
Insomnia	Destabilizaition of the “sleep-wakefulness” switch	VLPO hypofunction	VLPO neurons remain active Wake structures fail to deactivate and sleep onset is impaired	Prolonged sleep latency Sleep fragmentation Difficulty falling and staying asleep Early awakenings
		Over-activity of orexin/ hypocretin system	Impaired LC modulation	
	Gene dysregulation	Dopaminergic pathway	CREB gene up-regulation	Sustained wakefulness
Narcolepsy	Hypocretin cell loss	VTA	REM-on firing	Premature entry into REMS Hallucinations Sleep paralysis Cataplexy
	MCH cell loss	Hypothalamus	REM-on firing	
RLS/PLM	Circadian rhythm dysregulation	RLS generator	RLS and PLM	Delayed sleep onset
	Circadian phase recovery	Neurotransmitter and hormonal fluxes	Hypersensitivity	Exaggerated hyperalgesia in feet Neuropathic pain
	Dopaminergic system dysfunction	A11-diencephalic dopaminergic nucleus Spinal tract and cord SCN Hypothalamus	Impaired sensory suppression Altered circadian rhythm regulation	Dysethesia
		Melatonin secretion dysregulation	Impaired sleep regulation	
	Dopaminergic drugs	Sensitivity changes in postsynaptic dopamine receptors	Impaired dopamine metabolism Response changes in prolactin and growth hormone	
RBD	Lewy body and neurite formation	REMS components dysfunction, including: SLD MCRF DRN Peri-LC (reduced monoaminergic activity) Locomotor generator changes (inceasing activity in LDT and PPN)	Mood and behavior changes RWA	Vivid and frightening dreams Excessive muscle tone and activity Complex, dynamic and violent behaviors Dream enactment
	Dopaminergic dysfunction	VTA	Dysfunction in NRMC and coeruleus–subcoeruleus complex	
	Lesions	Ventral midbrain and medial medulla	Loss of muscle tone suppression	
Nocturia	Dopaminergic pathway dysfunction	D1 receptors (under-active) D2 receptors (over-active)	Detrusor over-activity	Frequent and excess urination at night
		SCN (Disrupted circadian control)	Nocturnal relaxation of bladder wall Increased urethral sphincter tone (abnormal bladder contraction and urethral sphincter relaxation)	
SAS	Nocturnal akinesia/ dyskinesia of respiratory muscle	Upper airway muscle dysfunction	Vocal cord abductior dysfunction	Loud crescendo snoring Irregular snorting Gasping Nocturnal stridor
	Dopaminergic durg excess and withdrawal (hypersensitive dopamine receptors)	Oromandibular/ laryngeal dystonia Chest wall muscle bradykinesia and rigidity	Respiratory compromise	Dyspnea Tachypnea Irregular, erratic breathing patterns

## Sleep disorders in Parkinson's disease

### Excessive daytime sleepiness (EDS)

EDS is a disabling trend of rapid sleep onset without prior drowsiness in various circumstances (Frucht et al., 1999) It is a marker of dopamine loss and the first consequence of dopamine deficiency prodromal to PD (Arnulf et al., 2002). It is associated with severe PD, PD-related disability, cognitive decline, frequent hallucinations, dementia and extended drug therapy including antihistamines, dopaminergic therapy, anxiolytics and SSRIs levodopa and dopamine agonist therapy (Dhawan et al., 2006; Comella, 2007; Belaid et al., 2014).

EDS in PD is mainly due to arousal system damage, especially owing to selective orexinergic neuronal loss in the posterior lateral hypothalamus innervating central targets in advanced PD stages (Arnulf, 2005). This causes a reduction in the number of A10 dopaminergic neurons in the VTA (Rye, 2004) as orexin yields Hcrt. This further promotes wakefulness by up-regulating the mono-aminergic neuronal population. Thus balance is more in favor of REM-on firing and wakefulness is impaired, which can lead to narcolepsy (Siegel, 2009; Suzuki et al., 2011).

Supplementary to orexin and histamine systems, impairments in serotoninergic, noradrenergic, and cholinergic neurons in the brainstem which serve as arousal systems that maintain wakefulness also result in EDS (Suzuki et al., 2011).

Dopaminergic drugs are used to ameliorate EDS (España and Scammell, 2011). DA agonists however have a sedative effect due to differential selectivity for D1 and D2 receptors. D1 agonists and small doses of DA have been shown to increase firing of orexin neurons in the rat hypothalamus, while high concentrations of DA and D2 agonists decrease block this firing (Alberto et al., 2006). Thus patients with partial orexin deficiency due to low catechol o-methyl-transferase and monoamine oxidase inhibitors (MAO-I) activity would be sedated by D2–D3 agonists or high doses of levodopa due to prolonged action of DA in the synaptic cleft (Arnulf, 2005).

### Insomnia

Insomnia exhibits prolonged sleep latency and fragmentation characterized by difficulty in falling and staying asleep, early awakening, or non-refreshing sleep despite adequate opportunities for sleep, coupled with impaired daytime functioning (Suzuki et al., 2011).

**Figure 7:**
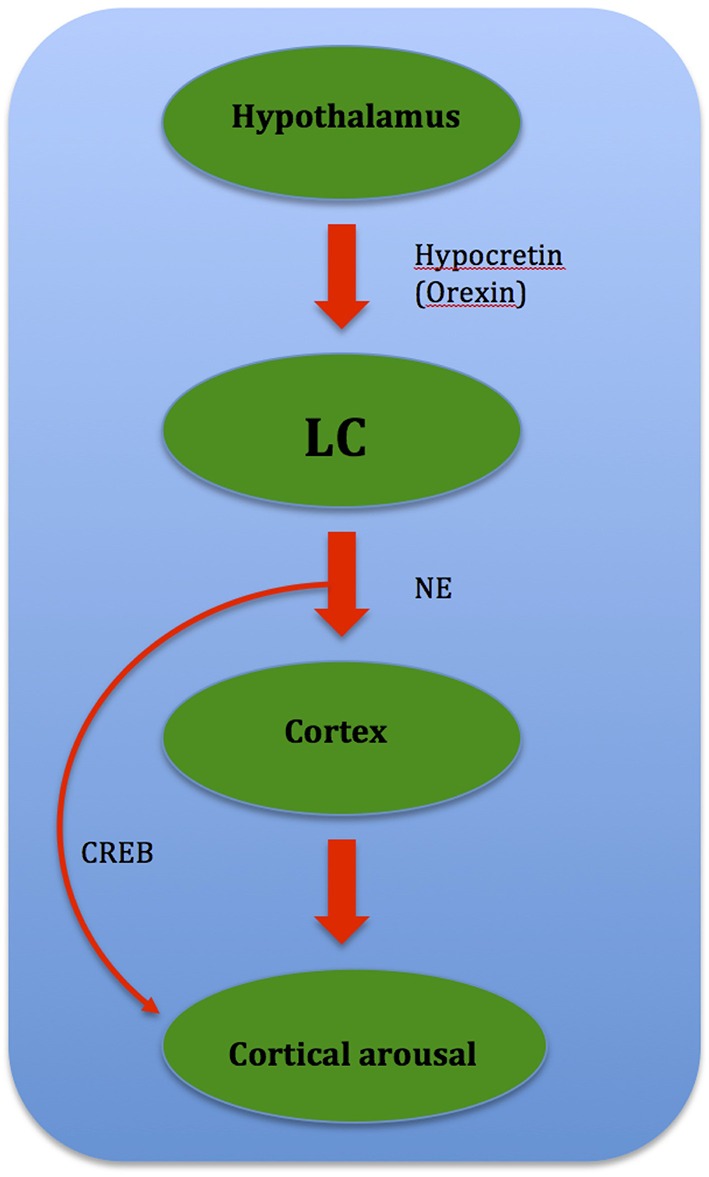
**Flow chart showing relationship between brain structures governing cortical arousal: CREB (cAMP-response element binding protein) mediates cortical arousal when prompted by NE signals from the LC**.

Insomnia occurs when there is a destabilization of the “sleep-wakefulness switch,” either through altered neurotransmitter release via medication or by a morphological lesion or inflammation. This is hypothesized to cause imbalance between sleep-promoting and arousing brain areas, leading to a hypofunction of the VLPO and/or an over-activity of the Hcrt system (Riemann et al., 2010). This results in insomnia as wake structures fail to deactivate during the transition from wake to sleep. Up-regulation of the LC and other arousal strucutres via the orexin/ Hcrt system (Aston-Jones et al., 2001) leads to insomnia as this promotes arousal due to over-activity (Figure 7).

**Figure 8:**
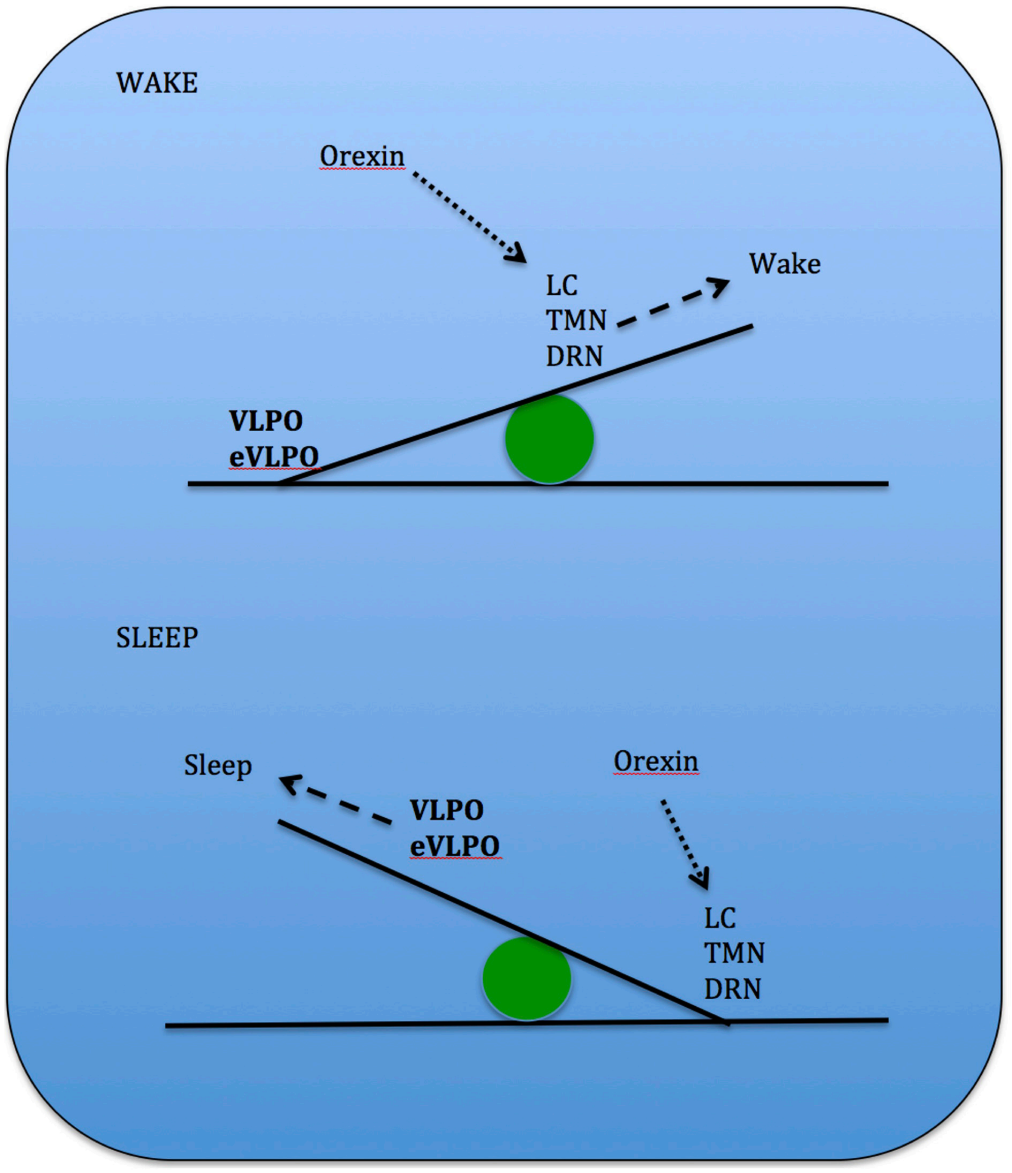
**Key brain structures affected by Lewy bodies, Lewy neuritis, and neuronal loss n Parkinson's disease (highlighted in red box): SN, substantia nigra; DRN, dorsal raphe nuclei; vlPAG, ventrolateral periaqueductal gray; LDT, laterodorsal tegmental nucleus; PPN, pedunculopontine nucleus; LC, locus coeruleus; SLD, sublaterodorsal nucleus; MCRF, magnocellular reticular formation**. (Adapted and published under the Creative Commons Attribution-Share Alike 3.0 Unported license).

Gene dysregulation is also speculated to cause insomnia in PD. One such gene is CREB, or cAMP-response element binding protein, where manipulations of CREB conducted through molecular genetic techniques in mice suggest that CREB is involved in sustained arousal (Graves et al., 2003). The arousal-promoting areas, especially the LC, which influences wake-promoting neuronal groups through the cAMP/PKA/CREB signaling pathway activation plays a possible role. Reciprocally after phosphorylation, CREB induces gene expressions that help sustain wakefulness (Cirelli and Tononi, 2000), and may mediate cortical arousal in response to NE signals from the LC (Graves et al., 2003). DA is known to play a role in CREB gene phosphorylation, and it was shown that loss of dopaminergic input leads to an oversensitivity of striatal neurons toward DA-induced CREB phosphorylation than normal (Cole et al., 1994). Thus it is possible that such a phenomenon leads to CREB gene up-regulation, which results in gene expressions that sustain wakefulness. CREB may also mediate cortical arousal in response to NE signals from the LC, as recent data (Aston-Jones et al., 2001; Sutcliffe and de Lecea, 2002) suggest that the orexin/ Hcrt system regulate the activity of the LC (Graves et al., 2003). This in turn leads to CREB gene up-regulation and thus sustained wakefulness (Figure 8).

### Narcolepsy

Narcolepsy occurs when there is abnormal short delay and premature entry into REMS, due to disconnections between REMS components which lead to waking (Carskadon and Dement, 2011). This results in hypnagogic hallucinations, sleep paralysis, and most dramatically, cataplexy.

Pathophysiology is linked to Hcrt neurons carrying hypothalamic peptides mediating wakefulness via projections to the VTA, which were almost undetectable in narcoleptic patients (Chaudhuri and Schapira, 2009). Thannickal et al. also showed that PD patients endeavor progressive Hcrt cell loss with advancing disease, along with melanin-concentrating hormone cell loss throughout the anterior and posterior hypothalamus. This occurs even before dopaminergic cell loss which result in motor symptoms. Early loss transpiring before drug treatment not only explains narcolepsy but also orthostatic hypotension in PD and abnormal body temperature regulation (Thannickal et al., 2007) owing to impaired hypothalamic function. As mentioned above, Hcrt promotes wakefulness by up-regulating the mono-aminergic neuronal population. As PD patients are observed to have almost complete loss of Hcrt cells, the balance is more in favor of REM-on firing and thus wakefulness is impaired, leading to narcolepsy (Siegel, 2009; Suzuki et al., 2011). It is to be noted that this phenomenon occurs simultaneously while dysregulation of DA, NE, and SE occur at the same time, hence further impairing arousal mechanisms and inducing sleep.

### Restless legs syndrome (RLS) and periodic limb movements (PLM)

RLS is manifest by the need to move due to dysethesias in the limbs that transpire at rest, and is eased by movement. Symptoms often occur in a circadian pattern, beginning in the day during periods of rest and becoming more intense in the evening/night (Baier and Trenkwalder, 2007). The circadian pattern of periodic limb movements (PLM) that occur almost analogously could be secondary to the phasic influence of RLS (Lewy, 1999). Sensory symptoms and PLM during wakefulness further prevent sleep onset.

Although the central circadian pacemaker does not exhibit any abnormalities, the severity of symptoms might be indirectly modulated by an underlying circadian variation. RLS presents an exacerbation of symptoms at night, supporting the factor of circadian oscillation. In a study conducted to address circadian symptom modulation, it was found that circadian oscillation of motor and sensorial symptoms can be observed under conditions of sleep deprivation as well. This suggests homeostatic sleep drive as an additional factor modulating RLS symptoms. Observations of RLS seen to be most intense late in the circadian period, and least intense several hours later early in the following circadian period suggests the presence of a generator for RLS. PLM is also observed to occur simultaneously, suggesting the circadian pattern of PLM to be secondary to phasic influence from the RLS generator (Lewy, 1999).

Exaggerated hyperalgesia in the feet, (Baier and Trenkwalder, 2007), and neuropathic pain were also shown to be worse in the evening and night (Odrcich et al., 2006). This evidences that both circadian phase and recovery following nocturnal sleep play a role in pain sensitization, thus postulating that endogenous fluxes in neurotransmitters and hormones succeeding circadian phases are involved in these variations (Baier and Trenkwalder, 2007).

The pathophysiology of brain structures involved have not been studied extensively, however animal (Perlow et al., 1977; Schade et al., 1995) and human studies (Sowers and Vlachakis, 1984; Doran et al., 1990) advocate the existence of circadian variations in dopaminergic activity (Andretic and Hirsh, 2000; Nir et al., 2000). Human data show a distinct circadian variation, with a pattern characterized by an increase in the morning and a nadir in the late evening/night (Winkelman, 2006). Furthermore, circadian variation in the DA system also influences melatonin secretion, thus affecting sleep regulation. However, the circadian pattern might not be generated by the dopaminergic system itself, but by other factors that indirectly modulate it.

RLS secondary to PD has demonstrated that both these diseases can be treated by specific dopaminergic medications where both diseases exist (Winkelman, 2006; Möller et al., 2010). Thus dopaminergic system dysfunction in RLS pathophysiology is most probably caused by impaired central dopaminergic transmission (Trenkwalder and Paulus, 2004; Winkelman, 2006), which is also the cause for PD symptoms. Pathophysiology is linked to the A11-diencepalic dopaminergic nucleus which provides the main descending dopaminergic control of the spinal tract (Qu et al., 2006), serving as a relay to the spinal cord due to close anatomical relationship with the SCN in the hypothalamus (Abrahamson and Moore, 2001) involved in circadian rhythm regulation. A11 cell bodies also project into the dorsal horns and intermediolateral tracts of the spinal cord (Björklund and Skagerberg, 1979; Albanese et al., 1985), which are involved in sensory suppression (Trenkwalder and Paulus, 2004). Therefore, dopaminergic dysfunction in this area might contribute to the sensory symptoms of RLS.

Neuroendocrine responses to dopaminergic drugs also contribute to the cause of RLS. Response changes in prolactin and growth hormone release after L-dopa administration were observed at night but not during the day in RLS patients. This indicates a consequence of circadian changes in the sensitivity of postsynaptic dopamine receptors at night (Garcia-Borreguero et al., 2004), exhibiting impaired dopamine metabolism (Baier and Trenkwalder, 2007). Thus it is suggestive that there is an increase in the amplitude of circadian variation of dopaminergic function in patients with RLS compared to healthy controls.

### REMS behavior disorder (RBD)

RBD is characterized by vivid and frightening dreams or nightmares, associated with muscle activity that leads to dream enactment (Comella, 2007). EEG measurements confirm muscle activity due to lack of atonia, also known as REMS without atonia or RWA (Boeve et al., 2007). Patients experience drastic reductions in REMS, decreased sleep efficiency, and increased sleep fragmentation due to increased stage transitions and awakenings (Belaid et al., 2014). Abnormal periods of REMs also occur most often before the onset of REMS. Complex, dynamic, and violent behaviors due to dream enactment result in injuries in patients and their bed partners. Polysomnographic measurements also display excessive chin muscle tone and limb jerking during REMS (Comella, 2007).

**Figure 9:**
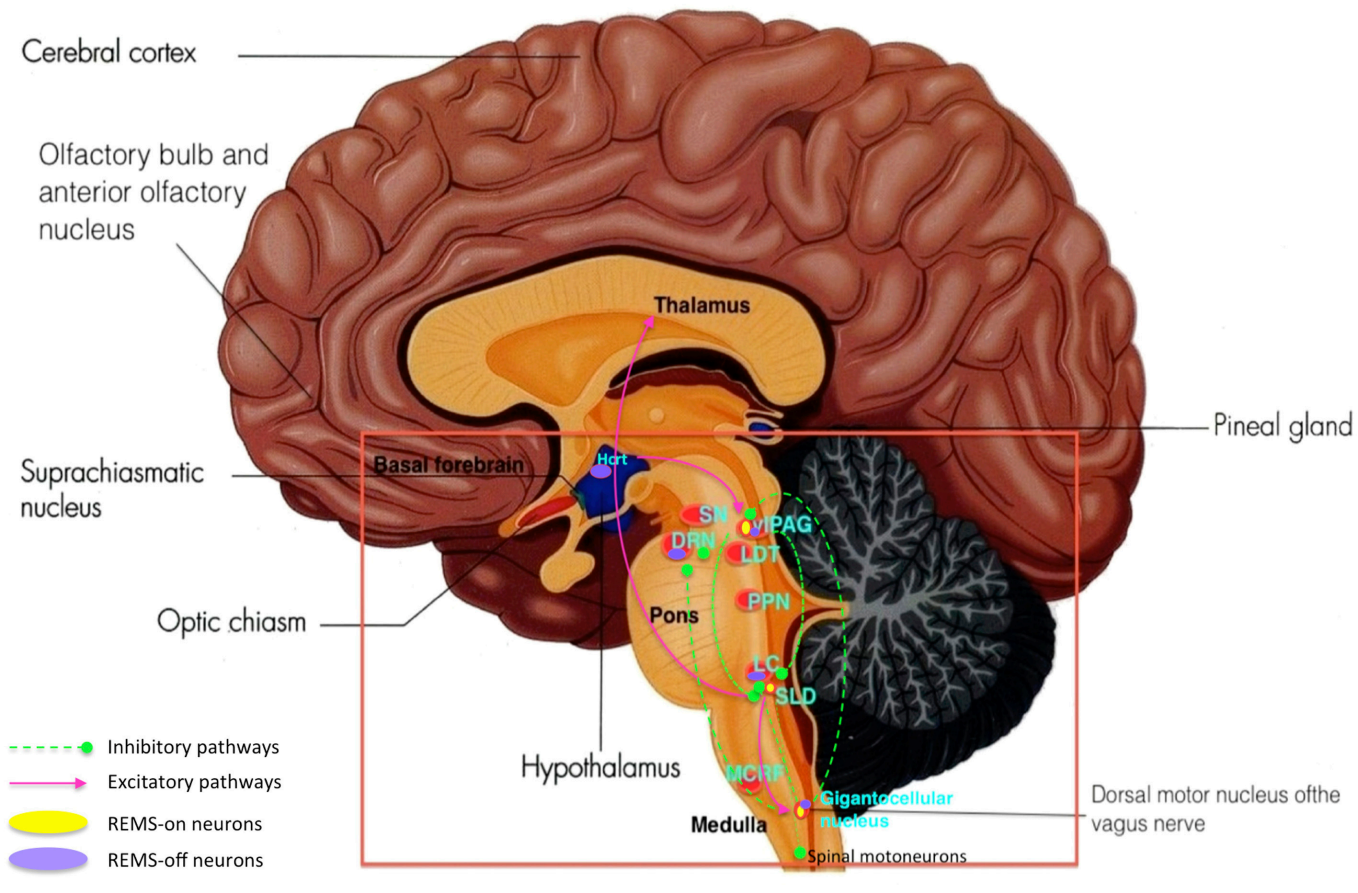
**Key brain structures affected by Lewy bodies, Lewy neuritis, and neuronal loss n Parkinson's disease (highlighted in red box): SN, substantia nigra; DRN, dorsal raphe nuclei; vlPAG, ventrolateral periaqueductal gray; LDT, laterodorsal tegmental nucleus; PPN, pedunculopontine nucleus; LC, locus coeruleus; SLD, sublaterodorsal nucleus; MCRF, magnocellular reticular formation**. REMS onset occurs when a certain balance is reached between REMS-on and off neurons as well as inhibitory and excitatory pathways within the different brain structures. Damage and degeneration to these brain structures and their relationships lead to RBD. (Adapted and published under the Creative Commons Attribution-Share Alike 3.0 Unported license).

RBD occurs when there is dysfunction in the SLD, magnocellular reticular formation (MCRF), and peri-LC structures, which further lead to RWA. Further degeneration of these structures, together with changes in locomotor generator structures, lead to obvious RBD. This chronological sequence of pathology explains why RBD precedes motor symptoms, cognitive decline and dementia in most patients who develop PD. In the Braak stage 2 description of PD, it is stated that further Lewy body and neurite formation in the structures involved in stage 1 appear, which are the dorsal motor nucleus of the vagus nerve, olfactory bulb and anterior olfactory nucleus complex. Lewy body and neurite formation begin to form in the SLD and precoeruleus region, the MCRF, the DRN, and LC. Once the accumulation threshold is reached, changes in mood, behavior, and sleep start to develop. Measurable changes in serotonin and noradrenaline might also be present at this stage. Degeneration in the SLD and precoeruleus region, MCRF, or both lessen the inhibitory effects on the caudal anterior horn cells, thus leading to RWA (Boeve, 2013) (Figure 9).

Based on animal studies, it is proposed that the SLD sends projections to spinal motoneurons through a direct route that causes active inhibition of skeletal muscle activity in REMS. The SLD also functions through an indirect route via the ventrolateral reticulospinal tract to reduce excitation of the MCRF, thus causing a net reduced inhibition of spinal motoneurons. Therefore lesions, degeneration and pharmacological manipulation of the SLD or MCRF can cause RWA, though not yet sufficient to cause dream enactment behavior (Boeve et al., 2007). The LC works together with other midbrain regions located beneath and lateral to the vlPAG (Suzuki et al., 2011). Due to dysfunction of the subcoeruleus nucleus controlling muscle atonia during REMS, reduced excitation of the MCRF and disinhibition of spinal motoneurons occur either directly or indirectly via other brainstem nuclei (Boeve et al., 2007).

It was also demonstrated that 2 systems are involved in normal REMS, where one generates muscle atonia and another suppresses locomotor activity. Muscle atonia is caused by active inhibition by the MCRF neurons in the medulla via the ventrolateral reticulospinal tract synapsing on spinal motoneurons. These MCRF neurons receive excitatory effects from the peri-LC region in the pons via the lateral tegmentoreticular tract. Neurons in the peri-LC region are thought to inhibit the LDT and cholinergic PPN, which is interconnected with the substantia nigra, hypothalamus, thalamus, basal forebrain, and frontal cortex. These pontine structures act as locomotor generators, and are believed to receive input from supratentorial structures, especially the forebrain and thalamus, which ultimately influence spinal motoneurons. Thus during REMS, phasic oculomotor and locomotor activity such as REMs and muscle twitches occur, however extravagant motor activity is directly or indirectly inhibited (Schenck et al., 1993). Therefore any changes especially to the peri-LC region, PPN, and LDT can also lead to RBD.

In relation to that, the first human RBD case ever reported presented a marked reduction in the number of neurons in the LC and increase in the number of neurons in the PPN and LDT (Schenck et al., 1996). Hence increased activity in cholinergic neurons and/or decreased disinhibition of the PPN and LDT due to reduced monoaminergic activity of the LC results in RBD. Other studies demonstrate that lesions extending from the ventral midbrain to the medial medulla also cause RWA as activation of this system suppresses muscle tone (Schenkel and Siegel, 1989; Lai and Siegel, 1990; Holmes et al., 1994; Lai et al., 1999, 2008).

Dopaminergic dysfunction in the VTA has also been associated with RBD pathophysiology based on anatomic (Rye, 1997), pharmacologic (Fantini et al., 2003), and functional neuroimaging studies (Albin et al., 2000; Johannes Schwarz and Noachtar, 2003). This further leads to dysfunction in the NRMC and coeruleus-subceruleus complex, thus leading to RBD.

### Nocturia

Nocturia is a condition of frequent and excess urination during the night. Autonomic dysfunction of the bladder leading to nocturia is a frequent complaint in PD patients, commonly among men (Cheon et al., 2008). Pathophysiology is linked to dopaminergic pathways affecting bladder activity as D1 receptors inhibit micturition reflex while D2 receptors activate it, therefore under-active D1 and an over-active D2 stimulation may explain this detrusor over-activity (Chaudhuri and Schapira, 2009).

The SCN also regulates osmotic pressure sensing cells responsible for nocturnal increase in arginine vasopression secretion, which subsequently reduces the volume of night-time urine production (Colwell, 2010; Trudel and Bourque, 2010). It is assumed that disrupted circadian control due to dopaminergic pathway dysfunction promotes nocturnal relaxation of the bladder wall and increased urethral sphincter tone. This leads to abnormal bladder contraction and relaxation of the urethral sphincter, thus increasing nocturia and urinary incontinence (Willison et al., 2013).

### Sleep apnea syndrome (SAS)

SAS results from a deficit in breathing drive in the brain (central sleep apnea), or a problem with airflow through breathing passages, also known as obstructive sleep apnea. As breathing becomes more difficult or ceases a decrease in blood oxygen level occurs, which in turn results in awakening to restore breathing. SAS leads to sleep disordered breathing, and is identified through a history of loud crescendo snoring and irregular snoring with snorting and gasping (Mitra and Chaudhuri, 2009). As the patient remains in light sleep, they may be unaware of these awakenings, which occur numerous times a night. Consequently, the patient experiences little deep restorative sleep at night which leads to EDS. Apnea has been found in as many as 50% of patients with PD. Snoring and apneic episodes may be up to three times more common in PD (12%) than in the general population.

Upper airway muscle dysfunction caused by nocturnal akinesia or dyskinesia of the respiratory muscle lead to development of obstructive sleep apnea, which develops onto life-threatening nocturnal stridor caused by vocal cord abductor dysfunction (Suzuki et al., 2011). This occurs due to suppression of SWS or REMS. Successful treatment of breathing problems with continuous nocturnal positive airway pressure produces large rebounds of SWS and REMS (Carskadon and Dement, 2011). Respiratory muscle dyskinesia occurs due to dopaminergic medication excess and withdrawal (Garcia-Borreguero et al., 2003). This results in dyspnea, tachypnea and irregular, erratic breathing patterns. Levodopa also induces oromandibular (Kato et al., 2007) or laryngeal dystonia (Onoue et al., 2003), causing respiratory compromise due to upper airway obstruction. These respiratory disorders are exhibited during the “on state,” and decline as medication effects cease. However, a wearing off effect can also be associated with respiratory complaints, particularly in advanced PD patients related to laryngeal dystonia, stridor, and appearance of chest wall muscle bradykinesia and rigidity (Hartman, 1988). Other wearing off symptoms compromising respiration includes shortness of breath which resembles a panic attack (Vázquez et al., 1993). The pathophysiology of levodopa-related respiratory problems is not well understood, but it is very likely associated to denervation hypersensitivity of dopamine receptors to exogenous dopamine in the peripheral chemoreceptor neurons (Rice et al., 2002). Dopamine is known to be involved in both peripheral chemoreceptor and brainstem respiratory center function as hypoxia increases the synthesis and release of endogenous dopamine by carotid body glomus cells (Iturriaga et al., 2009). Thus reduced peripheral chemosensitivity may explain the reduced ventilatory response to hypoxia in PD (Serebrovskaya et al., 1998).

## Conclusion

PD and its symptoms are caused by various dysfunctional structures compromising many control areas in the brain, resulting in motor and non-motor abnormalities including sleep. Good comprehension and knowledge of the various brain structures involved, their relationship, and their pathophysiology in the initiation and development of these symptoms will benefit in administering innovative treatments such as deep brain stimulation. Further studies focusing on genetics and biochemistry could also help in finding a possible cause and cure for this debilitating disease.

## Author contributions

Ms IF is the main author who wrote the manuscript in order to have a broader understanding on the pathophysiology of Parkinson's disease, especially in aspects Parkinson's disease and various brain structures in relation to sleep. KM was the author who supervised and helped in the writing of this manuscript.

### Conflict of interest statement

The authors declare that the research was conducted in the absence of any commercial or financial relationships that could be construed as a potential conflict of interest.

## Abbreviations

Electroencephalogram: EEG
Suprachiasmatic nucleus: SCN
serotonin: SE
dorsal raphe nuclei: DRN
norepinephrine: NE
ventral tegmental area: VTA
locus coeruleus: LC
pedunculopontine nucleus: PPN
non-rapid eye movement sleep: NREMS
rapid eye movement sleep: REMS
slow wave sleep: SWS
ventrolateral pre-optic nucleus: VLPO
gamma-aminobutyric acid: GABA
laterodorsal tegmental nucleus: LDT
sublateordorsal tegmental nucleus: SLD
ventrolateral periaqueductal gray: vlPAG
monoamine oxidase: MAO
autonomic nervous system: ANS
excessive daytime sleepiness: EDS; REMS behavior disorder
restless legs syndrome: RLS
periodic limb movements: PLM
REMS without atonia: RWA
magnocellular reticular formation: MCRF
sleep apnea syndrome: SAS.
